# Intelligent resource management in UAV-enabled networks using cell-free communications and intelligent reflective surfaces

**DOI:** 10.1038/s41598-026-43358-2

**Published:** 2026-03-10

**Authors:** Haiwei Wu, Fengrun Gu, Haifeng Lu, Hao Su

**Affiliations:** College of Information Engineering, Hainan Vocational University of Science and Technology, Haikou, 571126 Hainan China

**Keywords:** Reinforcement learning, UAV-assisted IoT, Intelligent reflecting surface (IRS), Cell-free networks, Energy science and technology, Engineering, Mathematics and computing

## Abstract

The swift proliferation of Internet of Things (IoT) applications necessitates wireless infrastructures that are scalable, energy-efficient, and able to satisfy rigorous quality-of-service (QoS) criteria. Traditional cellular networks frequently have difficulties in overcoming these obstacles due to restricted spectrum reuse, variable coverage, and elevated energy usage. Recently, unmanned aerial vehicles (UAVs) and intelligent reflecting surfaces (IRSs) have surfaced as potent alternatives for improving coverage and spectrum efficiency. This study presents a reinforcement learning (RL) optimization methodology for a cell-free UAV–IRS-supported IoT network. The suggested architecture enhances throughput, broadens coverage, and diminishes energy consumption through the optimization of UAV positioning, IRS phase modifications, and resource allocation. The efficacy of the proposed strategy is proved through a comparative analysis of its performance against multiple baseline methodologies. Comprehensive simulation findings indicate the superiority of the RL-based methodology, yielding a total throughput enhancement of 15.38% compared to the most effective baseline, augmenting coverage by 5.56%, and decreasing energy usage by 4.55%. The collaboration between UAVs and IRSs is emphasized, with UAVs offering flexible relaying and IRSs improving signal quality in resource-constrained settings. These findings confirm that reinforcement learning is an effective approach for managing next-generation UAV–IRS–cell-free IoT systems, providing substantial performance enhancements while maintaining sustainability and energy efficiency.

## Introduction

The swift expansion of IoT devices has fundamentally transformed wireless communications, requiring scalable infrastructures to provide ultra-reliable low-latency communication (URLLC) in many contexts^1,2^. In this changing environment, UAVs have become versatile facilitators, offering on-demand aerial communication to enhance coverage in areas where traditional terrestrial networks are inadequate, such as distant rural locations or disaster-affected regions^3^.

Simultaneously, cell-free communications signify a fundamental transformation from conventional cellular frameworks. Distributed access points (APs) cooperatively serve customers without defined cell boundaries, effectively reducing inter-cell interference and improving spectral efficiency^4^. Moreover, IRSs composed of programmable metamaterials provide a passive yet potent means to control signal transmission by dynamically modifying phase shifts, thus enhancing desired signals and mitigating interference^5^.

The combined utilization of UAVs, cell-free networks, and IRSs presents significant potential for future IoT ecosystems, where billions of devices necessitate uninterrupted connectivity under strict QoS requirements^6^. Hybrid UAV-enabled cell-free systems, enhanced by IRSs, are particularly essential for 6G wireless networks. They surmount the intrinsic constraints of static terrestrial infrastructure by utilizing UAV mobility to provide adaptable line-of-sight (LoS) connections, while IRSs facilitate passive beamforming to enhance energy efficiency and diminish the carbon footprint of wireless implementations^7^. This integration guarantees extensive coverage in key IoT applications such as smart agriculture, environmental monitoring, and industrial automation, effectively facilitating massive machine-type communications (mMTC), which are anticipated to constitute around 70% of future network traffic^8^.

Empirical evidence demonstrates that IRS-assisted UAV systems can enhance signal-to-noise ratios (SNRs) by 10–15 dB in obstructed situations, hence enabling reliable data transmission for energy-constrained IoT nodes^9^. Moreover, cell-free topologies facilitate equitable resource distribution, mitigating the coverage inconsistencies characteristic of traditional macro-cell architectures and enhancing fairness in diverse networks^10^. This technology convergence strongly coincides with the United Nations’ Sustainable Development Goals by facilitating robust communication infrastructures in underserved areas, hence improving disaster response and public safety^11^.

Notwithstanding these compelling benefits, UAV-enabled cell-free networks including IRSs encounter numerous ongoing hurdles. The dynamic movement of UAVs creates channel uncertainty and complicates resource allocation, frequently resulting in suboptimal throughput in multi-user environments^12^. Energy consumption constitutes a significant constraint; the finite battery life of UAVs demands effective energy harvesting methods, yet current protocols falter in reconciling energy harvesting and data transmission phases, especially when exacerbated by the complexities of IRS phase optimization^13^. Recent literature^14–17^ underscores the necessity for energy-conscious designs that can be seamlessly integrated into UAV-IRS setups.

Managing interference in cell-free environments is particularly challenging, as the lack of cell boundaries intensifies co-channel interference, particularly in dense IoT deployments with high spectrum reuse^18^. Although IRSs offer economical passive reflection, their fixed characteristics restrict flexibility in rapidly changing UAV-assisted environments, necessitating advanced real-time phase reconfiguration algorithms to mitigate route loss and fading^19^. Numerous current studies presume flawless channel state information (CSI), although actual faulty CSI can diminish performance by 20–30% in real-world scenarios^20^. Security weaknesses, including eavesdropping in open-air UAV communications, present considerable risks in multi-adversary IoT settings^21–23^. Regulatory limitations on UAV altitudes, coupled with scalability challenges in extensive networks (where simultaneous optimization of UAV positioning, IRS phases, and AP linkages becomes exponentially intricate), further hinder broad use^24,25^.

The complex challenges expose the shortcomings of conventional optimization techniques, such as outer approximation or evolutionary algorithms, which often converge to local optima and inadequately tackle the stochastic, high-dimensional dynamics of UAV-IRS-cell-free systems^26^. RL provides a robust, data-driven alternative that derives near-optimal policies via direct interaction with the environment, effectively addressing the multi-objective demands of IoT networks, such as throughput maximization, coverage improvement, and energy efficiency^27^. Recent improvements indicate that deep reinforcement learning approaches, including DDPG and its derivatives, provide real-time optimization of IRS phase shifts and UAV trajectories to enhance secrecy rates and energy efficiency in multi-user/multi-eavesdropper scenarios^21–23^.

The main aim of this project is to create a complete reinforcement learning-based framework that simultaneously optimizes UAV trajectories, IRS phase configurations, power allocation, and access point relationships in a cell-free IoT network. The architecture seeks to optimize overall throughput, enhance coverage equity for distributed IoT devices, and reduce total energy consumption, including UAV propulsion and IRS operations.

This work uniquely integrates time-switching (TS) energy harvesting at the UAV with IRS-enhanced relaying, conceptualized as a Markov decision process (MDP) within a multi-objective optimization (MOO) framework. The proposed method utilizes Q-learning with penalty-based reward shaping to adaptively address varying channel conditions, enforce QoS requirements, and attain balanced, near-optimal trade-offs, in contrast to previous systems that consider these factors in isolation.

This study presents the subsequent key contributions:

- A revolutionary system paradigm that combines cell-free architecture with UAV-IRS support, facilitating efficient simultaneous energy harvesting and data relaying for IoT devices in many situations, including urban obstructions and rural sparse deployments.
- A scalable reinforcement learning-based resource allocation method that diminishes computing complexity and accommodates large-scale networks via iterative Q-table updates and ε-greedy exploration.
- Comprehensive simulation results test the proposed framework against traditional methods, revealing enhancements of 15.38% in throughput, 5.56% in coverage, and 4.55% in energy efficiency, while clarifying the synergistic functions of UAV mobility and IRS passive beamforming.

The subsequent sections of this work are structured as follows. Section 2 examines pertinent literature and delineates significant research gaps that this study addresses. Section 3 delineates the suggested methodology, encompassing the system concept, TS relaying protocol, and MOO architecture. Section 4 assesses the framework’s efficacy using extensive simulations, emphasizing throughput, coverage, and energy usage measures. Section 5 examines the outcomes, focusing on computational complexity and comparative evaluation against benchmarks.

## Related studies

The swift advancement of IoT ecosystems has prompted extensive investigation into sophisticated wireless communication frameworks, especially those using UAVs, cell-free networks, and IRSs. These technologies tackle essential issues related to scalability, coverage, and energy efficiency for future networks. This section evaluates recent studies, highlighting their contributions, shortcomings, and the research gaps this work addresses.

Reference^28^ introduced a multicriterion resource management paradigm for energy-harvested cooperative UAV-enabled IoT networks, emphasizing power-splitting (PS)-based energy harvesting (EH) at UAV relays. Their methodology establishes a mixed-integer nonlinear programming (MINLP) problem to optimize the selection of IoT devices, assignment of UAV relays, allocation of source power, and PS ratio, with the objectives of maximizing network sum rate, enhancing device coverage, and minimizing carbon dioxide emissions. An outer approximation algorithm (OAA) was created, surpassing the nonlinear optimization methods of mesh adaptive direct search (NOMAD) and evolutionary algorithm (EA) regarding network utility and sum rate. Nonetheless, their research presupposes fixed UAV placements and excludes IRSs, hence constraining its flexibility in dynamic contexts and obstructed channels, which are essential in urban IoT implementations.

In^29^, an energy harvesting and information transmission scheme for a two-hop UAV relay system utilizing PS-based energy harvesting was examined. Their research presents an analytical solution for optimal power distribution in a multi-user system, enhancing energy efficiency under dynamic channel conditions by the application of the Lagrangian algorithm and the Lambert W function. The amalgamation of numerous antennas utilizing maximum ratio combining (MRC) improves signal dependability. Nonetheless, their design neglects the capacity of IRSs to alleviate interference and enhance link quality, and it lacks a cell-free architecture, hence limiting its applicability to extensive, scattered IoT networks.

In^30^, UAV-assisted wireless communications utilizing RF energy harvesting were examined, emphasizing trajectory optimization to improve throughput in IoT networks. Their modeling results indicate a 15% enhancement in data rate relative to fixed relay systems; however, the lack of IRS integration constrains performance in situations characterized by substantial path loss. Moreover, their dependence on heuristic optimization escalates computational complexity, presenting difficulties for real-time applications.

The research in^31^ presented a cell-free massive MIMO system, highlighting dispersed APs to improve spectral efficiency. The authors attained uniform coverage by zero-forcing beamforming; nevertheless, they neglected to address UAV relaying and energy harvesting, which are essential for enhancing connectivity in remote regions. The computational burden of their centralized processing restricts scalability in densely populated IoT systems.

In^32^, IRS-assisted wireless networks were examined, emphasizing the significance of phase shift optimization in enhancing SNRs by as much as 12 dB. The authors introduced a semidefinite relaxation method for configuring IRS elements while assuming static base station positions, overlooking the advantages of UAV mobility. This limits the framework’s adaptability in dynamic situations such as catastrophe recovery.

The study in^33^ integrated UAVs and IRSs inside a single-hop network, optimizing UAV positioning and IRS phase adjustments to enhance throughput. Although effective in small-scale configurations, their methodology fails to accommodate cell-free topologies or multi-objective optimization, hence constraining its capacity to balance coverage and energy efficiency in extensive IoT systems.

A RL-based solution for UAV trajectory optimization in cellular networks was developed in^34^, resulting in a 10% enhancement in throughput compared to conventional techniques. Nonetheless, the study excluded IRSs and EH, and its emphasis on cellular rather than cell-free topologies constrains its applicability to distributed IoT applications.

In^35^, a deep reinforcement learning framework for resource allocation in IoT networks was presented, maximizing power and spectrum utilization. Their findings indicate enhanced energy efficiency; nevertheless, they do not incorporate UAV or IRS integration, hence limiting their usefulness in situations necessitating airborne relaying or passive beamforming.

In^36^, a cell-free network with energy harvesting was examined, concentrating on power allocation to reduce energy usage. The lack of UAVs and IRSs, although successful in static configurations, restricts its capacity to manage dynamic channel circumstances and coverage deficiencies in obstructed situations.

In^37^, a MADDPG-based framework for UAV trajectory control in MEC systems was suggested, resulting in a 25–30% decrease in UE energy consumption and up to a 90% enhancement in fairness indices for job offloading. In^38^, low-complexity SCA algorithms for multi-UAV-assisted MEC in Industry 5.0 were introduced, aimed at lowering overall energy while adhering to URLLC limitations. Although these methodologies enhance energy-efficient and equitable UAV coordination, they predominantly focus on task offloading and MEC contexts, neglecting cell-free large access and IRS-assisted passive beamforming. This presents considerable opportunities for collaborative reinforcement learning-based optimization of UAV positioning, IRS phases, and resource allocation in extensive IoT networks, as discussed in this article.

The amalgamation of IRS with novel communication modalities has also garnered momentum. For example^39^, investigated a computational framework for RIS-augmented backscatter and direct communication in 6G IoT. Utilizing the Deep Deterministic Policy Gradient (DDPG) algorithm, they tackled issues including incomplete channel state information (CSI) and hardware constraints, markedly enhancing secrecy rates and energy efficiency. Likewise, the utilization of these technologies has been expanded to extraterrestrial platforms. Reference^40^ examined a secure multi-user satellite downlink system augmented by hybrid RIS. Their research illustrates that the joint optimization of satellite and RIS beamforming by deep reinforcement learning may surmount the large dimensionality of these systems, establishing a benchmark for hybrid-RIS performance compared to conventional passive designs.

The research in^41^ investigated IRS-assisted UAV communications, revealing a 20% enhancement in coverage probability by phase shift optimization. Nevertheless, it depended on optimal CSI, which is unfeasible in practical environments with defective CSI, and did not account for cell-free topologies.

A multi-objective optimization approach for UAV-enabled networks was suggested in^42^, balancing throughput and energy efficiency. The authors employed an evolutionary approach but neglected IRS contributions and cell-free topologies, leading to inferior performance in dense IoT environments.

Ultimately, in^43^, a hybrid UAV-IRS system was examined, emphasizing energy-efficient relaying. The authors attained a 15% decrease in energy usage; however, they did not incorporate reinforcement learning or cell-free topologies, hence constraining adaptability and scalability.

These studies collectively highlight significant shortcomings: most frameworks concentrate on UAVs, IRSs, or cell-free networks in isolation, neglecting their potential for synergy. Furthermore, few incorporate reinforcement learning for dynamic resource management, and many assume optimal conditions, disregarding practical constraints such as insufficient channel state information or restrictions in UAV mobility.

For handling constraints in RL, dynamic penalty approaches, such as those proposed in^44^, gradually increase penalty factors during training to enforce feasibility while improving convergence. This study presents a reinforcement learning framework that integrates UAVs, IRSs, and cell-free architectures to enhance throughput, coverage, and energy efficiency through a time-switching energy harvesting protocol and multi-objective optimization, offering a scalable and practical solution for IoT networks.

## System model and problem formulation

**Fig. 1:**
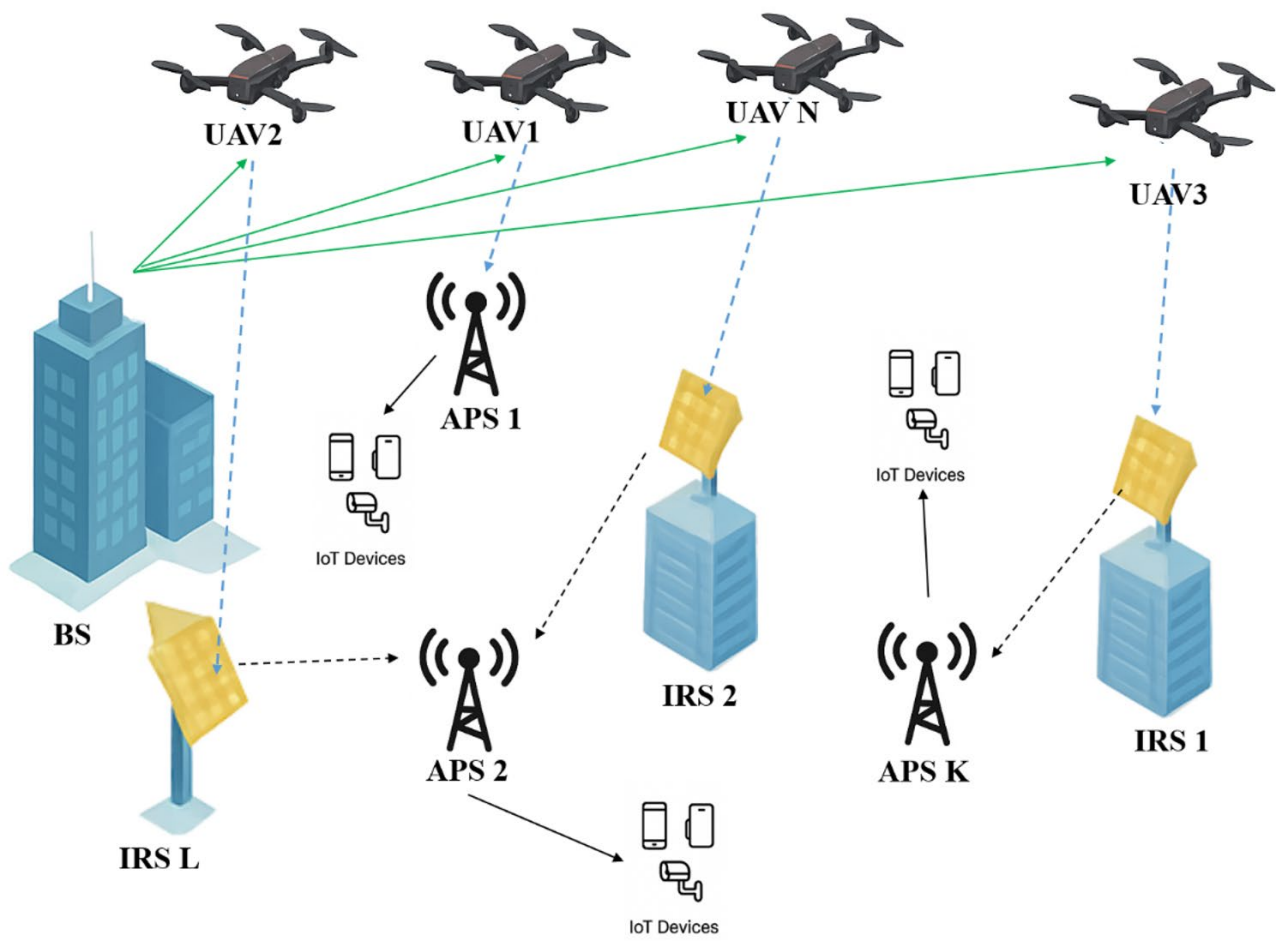
Conceptual view of the proposed network model.

This section delineates the system architecture for an intelligent UAV-enabled network augmented by cell-free communications and IRS to optimize resource allocation, as seen in Fig. 1. The proposed system architecture for intelligent resource management in UAV-enabled cell-free networks with IRS is depicted, demonstrating the integration of a central base station (BS), multiple UAV relays, distributed APs, and static IRS units to facilitate IoT devices. The network consists of a central BS, dispersed APs, UAVs functioning as aerial relays, IRS units with individual reflecting elements, and IoT devices, all outfitted with numerous antennas to utilize spatial diversity. The base station transmits RF signals for data transmission and energy harvesting, while unmanned aerial vehicles function as aerial relays utilizing a TS decode-and-forward (DF) protocol. The IRS units dynamically modify phase shifts to improve signal transmission and reduce interference, hence assuring equitable resource allocation within the cell-free framework. The access points, interconnected in a cell-free configuration, guarantee consistent coverage and uninterrupted connectivity for distributed IoT devices. The goal is to develop a multi-objective optimization problem that simultaneously optimizes access point selection, unmanned aerial vehicle trajectories, power distribution, and intelligent reflecting surface phase configurations, emphasizing the collaborative function of UAVs and IRSs in enhancing throughput, coverage, and energy efficiency. The proposed system is engineered to function efficiently across diverse practical situations. In urban settings, characterized by a high concentration of IoT devices and intricate signal propagation issues caused by structures and barriers, IRS units can dynamically modify phase shifts to reduce interference and improve signal strength, while UAVs offer adaptable aerial coverage to circumvent obstacles. In rural or remote regions, where conventional infrastructure is limited, the cell-free architecture integrated with UAV relays guarantees extensive coverage and connectivity for dispersed IoT devices, including agricultural sensors and environmental monitoring nodes. In disaster recovery situations, UAVs with energy collecting capabilities can be swiftly deployed to reestablish network connectivity, while IRS units improve signal dependability in difficult terrains.

Table 1 offers an exhaustive compilation of symbols and variables employed in the mathematical formulas within this section to improve reading.

**Table 1:** Symbol Definitions.

Symbol	Description
${P}_{s}$	Transmit power of the base station (BS)
${{\upeta}}_{n}$	Energy harvesting efficiency at UAV n
${{\uptau}}_{n}$	Time-switching factor for energy harvesting and relaying at UAV n
${{\Phi}}_{l}$	IRS phase shift matrix for IRS l
${{\upvarphi}}_{i}$	Phase shift of the i-th element of IRS
${h}_{opt,s,n}$	Effective channel gain from BS to UAV n, enhanced by IRS
${h}_{direct,s,n}$	Direct channel gain from BS to UAV n
${h}_{IRS,s,n,l}$	Channel gain from BS to IRS l for UAV n
${h}_{IRS,n,l}$	Channel gain from IRS l to UAV n
${h}_{s,m,k}$	Channel gain from BS to IoT device m for AP k
${h}_{opt,n,k}$	Effective channel gain from UAV n to AP k, enhanced by IRS
${\sigma}_{k}$	Noise power at AP k
${\sigma}_{s,n}$	Noise power at UAV n during EH phase
${\sigma}_{n,k}$	Noise power at AP k during relaying phase
${I}_{int,k}$	Interference power at AP k
${{\upbeta}}_{n}$	Dynamic power allocation factor for relaying at UAV n
${E}_{n,H}$	Harvested energy at UAV n
${P}_{n,k}$	Relaying power allocated to AP k from UAV n
${R}_{k}^{CF-base}$	Baseline cell-free rate for AP k
${R}_{k,n}^{CF-relay}$	Relayed rate from UAV n to AP k, enhanced by IRS
${R}_{k,n}^{CF-total}$	Total cell-free rate for AP k combining baseline and relayed contributions
${{\upgamma}}_{opt,s,n}$	Effective SNR during the EH phase for UAV n
${{\upgamma}}_{n,k}$	Effective SNR during the relaying phase from UAV n to AP k
${P}_{1},{P}_{2},{P}_{3}$	Penalty coefficients for multiobjective optimization
${\upalpha}$	Binary AP selection vector
${\upgamma}$	Binary UAV/IRS assignment matrix
K	Number of access points (APs)
N	Number of UAVs
L	Number of IRS units
M	Number of IoT devices
T	Total time frame for TS protocol
F(x)	Penalty-based objective function for multiobjective optimization
${f}_{throughput}$	Objective function for cell-free throughput
${f}_{coverage}$	Objective function for AP coverage
${f}_{energy}$	Objective function for energy consumption
${{\upzeta}}_{n,k}$	Auxiliary variable representing relayed rate contributions
$\epsilon$	Convergence threshold for Q-learning and relayed rate stability
${{\upalpha}}_{\mathrm{RL}}$	Learning rate in Q-learning (to distinguish from AP selection vector)
${{\upgamma}}_{\mathrm{RL}}$	Discount factor in Q-learning (to distinguish from UAV/IRS assignment matrix)
$r\left(s,a\right)$	Reward function in reinforcement learning

### Channel model details

This work models communication channels utilizing Rician fading to incorporate both line-of-sight (LoS) and non-line-of-sight (NLoS) signal components. The Rician K-factor, denoting the ratio of power in the LoS component to that in the scattered components, is a crucial element in our model. The K-factor is essential for assessing the signal intensity at the receiver, with common values of K set at 10 dB for urban settings and 20 dB for rural or line-of-sight conditions. To precisely represent the practical limitations of cell-free networks with mobile aerial nodes, we integrate the effects of incomplete Channel State Information (CSI). This enables the model to consider the overhead of Channel State Information acquisition and the possibility of pilot contamination, which are intrinsic to settings characterized by high mobility and restricted orthogonal pilot sequences.

We utilize the usual path-loss model for free-space line-of-sight communication for air-to-ground interactions between UAVs and the base station. The route loss is determined as follows:

1 $$PL\_LoS=20log_{10}\left(d\right)+20log_{10}\left(f\right)+C$$

where $d$ is the distance between the UAV and the ground station, $f$ is the carrier frequency, and $C$ is a constant dependent on the units.

The path-loss model for IRS-assisted communication includes the distance between the UAV and the IRS, together with the IRS’s reflection and refraction effects. The IRS phase shifts are designed to enhance signal propagation. The effective channel gain from the UAV to the IRS is denoted as:

2 $${h}_{IRS}={h}_{direct}+{\sum}_{l=1}^{L}\left({h}_{IRS,l}\cdot{{\Phi}}_{l}\cdot{h}_{IRS,n,l}\right),$$

where ${\Phi}_{\mathrm{l}}$ represents the phase shift matrix for each IRS element, and ${h}_{IRS,l}$ signifies the channel gain from the IRS unit to the UAV. The architecture improves signal dependability by utilizing the passive beamforming capabilities of the IRS, especially in situations when pilot contamination may compromise the quality of direct links.

Small-scale fading is integrated via Rayleigh fading for NLoS scenarios and Rician fading for LoS scenarios, resulting in stochastic fluctuations in signal strength influenced by the ambient environment. Moreover, the model incorporates the effects of UAV jitter and swift channel alterations by integrating these temporal fluctuations into the small-scale fading parameters. These variations are especially significant in urban and obstructed settings, when the UAV’s line of sight to the base station or IRS may be partially impeded. The suggested channel model incorporates the stochastic characteristics of mechanical jitter, ensuring that the IRS phase alignment remains resilient under real-time airborne disturbances.

The proposed channel model, by incorporating small-scale fading, quick fluctuations, and estimation inaccuracies, establishes a solid basis for the RL agent to develop resource allocation methods that reduce signaling overhead while preserving high spectral efficiency.

### Time-switching relaying model with IRS enhancement

The relaying model integrates RF energy harvesting (EH) at UAVs, enhanced by IRS-induced channel gains. The signal received at UAV n is analyzed during a time-switching phase ${{\uptau}}_{n}\in\left[\mathrm{0,1}\right]$ for energy harvesting with efficiency ${\eta}_{n}\in\left[\mathrm{0,1}\right]$, followed by data decoding and relaying in the remaining time $1-{\tau}_{n}$. The IRS utilizes a phase matrix ${{\Phi}}_{l}=\mathrm{diag}\left({e}^{j{{\upvarphi}}_{1}},\dots,{e}^{j{{\upvarphi}}_{M}}\right)$to optimize the effective channel gain. Harvested energy facilitates UAV operations, utilizing a dynamic power allocation factor ${\upbeta}$_n for relaying purposes. The time frame T is segmented into EH and transmission phases, optimized for energy economy.

The harvested energy at ${UAV}_{n}$ is given by:

3 $${E}_{n,H}={\eta}_{n}{\tau}_{n}\mid{h}_{opt,s,n}{\mid}^{2}{P}_{s}T$$

where ${h}_{opt,s,n}$is the effective channel from the BS to ${UAV}_{n}$ enhanced by IRS, and ${P}_{s}$is the BS transmit power.

The relaying power allocated to ${AP}_{k}$ is expressed as:

4 $${P}_{n,k}={\beta}_{n}{\eta}_{n}{\tau}_{n}\mid{h}_{opt,s,n}{\mid}^{2}{P}_{s}/(1-{\tau}_{n})$$

The fundamental cell-free rate for $A{P}_{k}$, accounting for inputs from all IoT devices, is:

5 $${R}_{k}^{CF-base}={\mathrm{l}\mathrm{o}\mathrm{g}}_{2}(1+{\sum}_{m=1}^{M}\mid{h}_{s,m,k}{\mid}^{2}{P}_{s}/{\sigma}_{k}^{2})$$

The relayed rate from ${UAV}_{n}$ to ${AP}_{k}$, enhanced by IRS, is:

6 $${R}_{k,n}^{CF-relay}=(1-{\tau}_{n}){\mathrm{l}\mathrm{o}\mathrm{g}}_{2}(1+\mid{h}_{opt,n,k}{\mid}^{2}{P}_{n,k}/({\sigma}_{n,k}^{2}+{I}_{int,k}\left)\right)$$

The aggregate cell-free rate, encompassing both baseline and relayed contributions, is:

7 $${R}_{k,n}^{CF-total}={R}_{k}^{CF-base}+{R}_{k,n}^{CF-relay}$$

The IRS phase matrix ${{\Phi}}_{l}$is optimized to maximize the effective channel gain, subject to unit modulus constraints:

8 $${{\Phi}}_{l}=\mathrm{a}\mathrm{r}\mathrm{g}{\mathrm{m}\mathrm{a}\mathrm{x}}_{{\Phi}}{\mid{h}_{opt,n,k}\mid}^{2}\mathrm{s.t.}\mid{\varphi}_{i}\mid=1,\forall i$$

The effective SNR during the EH phase is defined as:

9 $${\gamma}_{opt,s,n}={\tau}_{n}\mid{h}_{opt,s,n}{\mid}^{2}{P}_{s}/{\sigma}_{s,n}^{2}$$

And the effective SNR during the relaying phase is:

10 $${\gamma}_{n,k}=\mid{h}_{opt,n,k}{\mid}^{2}{\beta}_{n}{\eta}_{n}{\tau}_{n}\mid{h}_{opt,s,n}{\mid}^{2}{P}_{s}/\left[\right(1-{\tau}_{n}\left)\right({\sigma}_{n,k}^{2}+{I}_{int,k}\left)\right]$$

The network configuration exhibits a cell-free topology comprising K APs, N mobile UAVs, L static IRS units, and M IoT nodes, wherein BS transmission of RF signals facilitates data provision and enables energy harvesting. The efficient channel is represented as

11 $${h}_{opt,n}={h}_{direct,s,n}+{\sum}_{l=1}^{L}{h}_{IRS,s,n,l}{{\Phi}}_{l}{h}_{IRS,n,l}$$

${{\Phi}}_{l}$ aligns phases for optimal gain. The TS protocol designates ${{\uptau}}_{n}$ T for energy harvesting and $\left(1-{{\uptau}}_{n}\right)$T for relaying, with IRS-enhanced signal-to-noise ratio augmenting overall performance.

### Multi objective optimization framework

This subsection presents a MOO framework for the intelligent management of network resources. The primary objectives are to maximize cell-free throughput, enhance AP coverage, and minimize energy consumption, encompassing UAV propulsion and IRS operation.

A penalty-based approach is utilized to impose trade-offs via dynamically modified penalty coefficients P₁, P₂, and P₃, which impose penalties for departures from optimal objective values. The optimization problem is addressed by Q-learning, which is appropriate for scenarios with discrete action spaces and controllable state spaces, particularly when computational resources are constrained. Unlike Deep Q-Networks (DQN), which necessitate intricate neural networks and entail significant processing costs, Q-learning offers a more straightforward and effective resolution to this issue. The discretization of actions and states enables Q-learning to sustain a minimal computing load while efficiently optimizing resource allocation.

The decision variables consist of ${P}_{s}$ (base station power), ${\tau}_{n}$ (time slot factor), α (binary AP selection vector), and γ (binary UAV/IRS assignment matrix). The problem is formulated as a MINLP problem, subject to constraints on QoS, interference limits, and UAV mobility, specifically C1 to C10, which include minimum data rate, interference threshold, unit phase shifts, and UAV flight range. The overall optimization problem is articulated as

12 $${\mathrm{m}\mathrm{i}\mathrm{n}}_{x}F\left(x\right)={P}_{1}[{R}_{max}-{f}_{throughput}(x\left)\right]+{P}_{2}[K-{f}_{coverage}(x\left)\right]+{P}_{3}{f}_{energy}\left(x\right)\mathrm{s.t.}{g}_{j}\left(x\right)\le0$$

where F(x) denotes the penalty-based objective function, and ${g}_{j}\left(x\right)$ signifies constraint functions. The purpose of cell-free throughput is

13 $${f}_{throughput}={\sum}_{k}{R}_{k,n}^{CF-total}$$

The AP coverage objective is

14 $${f}_{coverage}=\mid\alpha\mid$$

The energy consumption objective is

15 $${f}_{energy}={\sum}_{k}{E}_{BS,k}+{\sum}_{n}{E}_{UAV,n}+{\sum}_{l}{E}_{IRS,l}$$

The primary MOO problem is $\mathrm{MOOP}:$

16 $${min}_{\gamma,\tau,\alpha,P,{\Phi}}F\left(x\right)\:\mathrm{s.t.}\:C1\:\mathrm{to}\:C10$$

where $C1$ ensures a minimum data rate of 1 Mbps per AP (${R}_{k,n}^{CF-total}\ge1$ Mbps, $\forall k$), $C2$limits interference to below 10 dB (${I}_{int,k}\le10$ dB, $\forall k$), $C3$restricts phase shifts to unit modulus ($\mid{\varphi}_{i}\mid=1,\forall i,l$), $C4$caps UAV flight altitude at 200 m (${h}_{n}\le200$ m, $\forall n$), $C5$enforces power constraints (${P}_{s}\le20$ W), $C6$ensures ${\tau}_{n}\in\left[\mathrm{0,1}\right],\forall n$, $C7$requires unique UAV assignments (${\sum}_{k}{\gamma}_{n,k}\le1,\forall n$), $C8$maintains AP coverage within 500 m (${d}_{k,n}\le500$ m, $\forall k,n$), $C9$sets a maximum energy consumption of 50 Wh (${f}_{energy}\le50$ Wh), and $C10$ensures network connectivity (${\sum}_{n}{\gamma}_{n,k}\ge1,\forall k$). The extended MOO problem, incorporating relayed rate variables, is $\mathrm{MOOP-}\mathrm{ext}:$

17 $${\mathrm{m}\mathrm{i}\mathrm{n}}_{\gamma,\tau,\alpha,P,{\Phi},\zeta}F\left(x\right)\:\mathrm{s.t.}\:C1\:\mathrm{to}\:C12,{\zeta}_{n,k}={R}_{k,n}^{CF-relay}$$

where $C11$and $C12$extend constraints to relayed rate stability ($\mid{\zeta}_{n,k}-{R}_{k,n}^{CF-relay}\mid\le \epsilon$) and IRS phase synchronization (${{\Phi}}_{l}$ consistent across iterations), respectively. Auxiliary variables ${\zeta}_{n,k}$represent relayed rate contributions.

The constraint-handling mechanism in the proposed reinforcement learning algorithm (outlined in Algorithm 1) utilizes a dynamic penalty function approach^44^, whereby violations of constraints (C1–C10, encompassing minimum data rate, interference threshold, unit phase shifts, and UAV flight range) result in a progressive augmentation of the penalty coefficients Pi (e.g., Pi = Pi * ρ, where ρ > 1 is a scaling factor, such as 1.1). This adaptive strategy guarantees incremental enforcement of feasibility during training, akin to reward shaping methods that apply substantial negative rewards for infractions, thus directing the agent towards policies that satisfy constraints without early convergence. This approach enhances the accuracy of neural network approximations and expedites convergence to near-optimal solutions, as evidenced in vehicle control challenges^44^.

### Reinforcement learning approach

This article presents a RL methodology as the strategic resolution for the specified mixed-integer nonlinear programming (MINLP) issue, highlighting the achievement of near-optimal resource allocation in the cell-free UAV-IRS-assisted IoT network. The RL method frames the optimization challenge as a Markov Decision Process (MDP), wherein UAVs and IRSs operate as agents that acquire knowledge to enhance resource allocation via ongoing engagement with the dynamic network environment.

The state space is structured to comprehensively represent the dynamic conditions of the network while ensuring computational feasibility for tabular Q-learning. It encompasses efficient channel gains (${h}_{opt,s,n}$, ${h}_{opt,n,k}$) among the BS, UAVs, IRSs, and APs. The gains are initially normalized to the range [0, 1] using min-max scaling and subsequently discretized into a finite number of bins to ensure compatibility with the Q-table and to prevent excessive expansion in state dimensionality. The positions of UAVs within the deployment area are normalized relative to the borders of the coverage region and discretized into a grid-based representation for each UAV, facilitating efficient encoding of mobility. The gathered energy levels at each UAV (${E}_{n,H}$) are normalized to the range [0, 1] according to the maximum potential harvested energy and quantized into discrete levels to accurately represent battery condition. The binary assignment matrices α (for AP selection) and γ (for UAV-to-AP and UAV-to-IRS assignments) are directly integrated as binary vectors, with improper configurations masked to significantly decrease the number of attainable states.

The action space comprises discrete modifications to the primary decision factors. This encompasses alterations to the base station transmit power ${P}_{s}$, incremental adjustments to the time-switching factors \tau_n that regulate the equilibrium between energy harvesting and data relaying at each UAV, toggling of access point selections within the binary vector α, and modifications to UAV/IRS assignment configurations in the matrix γ, along with enhancements to IRS phase shifts. To maintain controllable action dimensionality, IRS phase shifts are predominantly optimized according to Eq. (6), incorporating only essential discrete modifications as needed. Any activity that contravenes the problem constraints (C1–C10), including QoS thresholds, interference limitations, unit-modulus phase restrictions, or UAV mobility bounds, is obscured and disregarded, hence guaranteeing feasibility throughout the learning process.

The Q-learning algorithm incrementally develops and enhances a Q-table by assessing immediate and prospective rewards obtained from the multi-objective penalty function F(x) specified in Eq. (12). This reward framework is specifically crafted to concurrently enhance high throughput, extensive AP coverage, and minimal overall energy usage. The convergence of the learning process is established when the maximum variation in Q-values across all state-action pairs is below a specified stability threshold ε.

The standard Q-learning update rule is applied as follows:

18 $$Q(s,a)\leftarrow Q(s,a)+\alpha\left[r\right(s,a)+\gamma{\mathrm{m}\mathrm{a}\mathrm{x}}_{{a}^{{\prime}}}Q({s}^{{\prime}},{a}^{{\prime}})-Q(s,a)$$

where α is the learning rate, γ is the discount factor, and r(s, a) is the reward function, as detailed earlier.

The reward function is directly associated with the minimization of the penalty-based objective function F(x), as defined in the multi-objective framework:

19 $$r(s,a)=-F\left(x\right)$$

To tackle the dynamic equilibrium between optimizing total throughput and reducing energy consumption, specifically the mechanical and transmission energy of UAVs, the reward function is mathematically articulated as Eq. (20), which represents the penalty-based multi-objective optimization function delineated in Eq. (12):

20 $$F\left(x\right)={P}_{1}\left[{R}_{max}-{f}_{\mathrm{throughput}}\left(x\right)\right]+{P}_{2}\left[K-{f}_{\mathrm{coverage}}\left(x\right)\right]+{P}_{3}{f}_{\mathrm{energy}}\left(x\right)$$

Here, ${f}_{\mathrm{throughput}}\left(x\right)={\sum}_{k}{R}_{k,n}^{\mathrm{CF-total}}$ represents the aggregate cell-free throughput across all APs, which the framework aims to maximize; ${f}_{\mathrm{coverage}}\left(x\right)=\left|{\upalpha}\right|$ denotes the AP coverage (number of selected APs); and

21 $${f}_{\mathrm{energy}}\left(x\right)={\sum}_{k}{e}_{\mathrm{BS},k}+{\sum}_{n}{e}_{\mathrm{UAV},n}+{\sum}_{l}{e}_{\mathrm{IRS},l}$$

quantifies the overall energy consumption that the framework seeks to reduce. The reward feature is specifically designed to fundamentally alleviate the difficulties associated with CSI collection and pilot contamination. By integrating interference constraints as penalties into $F\left(x\right)$, the RL agent acquires the ability to circumvent resource allocations that would result in significant inter-UAV interference or excessive signaling overhead. This adaptive penalization guarantees that, despite pilot contamination prevalent in mobile cell-free situations, the agent chooses behaviors that sustain durable communication links and prioritize spectral efficiency over high-signaling configurations. Specifically, ${e}_{\mathrm{UAV},n}$ encompasses both mechanical propulsion energy, represented as

22 $${e}_{\mathrm{prop},n}={c}_{1}|{q}_{n}\left(t\right)-{q}_{n}\left(t-1\right){|}^{2}+{c}_{2}$$

where ${q}_{n}$ is the UAV position at time t, and ${c}_{1},{c}_{2}$ are constants based on UAV aerodynamics and hover power, and transmission energy,

23 $${e}_{\mathrm{trans},n}={\sum}_{k}{P}_{n,k}\left(1-{{\uptau}}_{n}\right)T$$

where T represents the time frame. The decrease in energy consumption attained by the RL agent also aids in diminishing the signaling overhead, since the policy promotes efficient, low-power trajectories that necessitate less frequent and less intensive CSI feedback in contrast to high-power, uncoordinated transmissions. ${R}_{max}$ is the theoretical maximum throughput, whereas K represents the total number of APs. The penalty coefficients ${P}_{1}$, ${P}_{2}$, and ${P}_{3}$ are dynamically modified during reinforcement learning training, specifically increased by a scaling factor $({\uprho}>1)$ in response to constraint violations, as outlined in the constraint-handling method, to enforce trade-offs. This formulation reconciles the objectives by imposing penalties for departures from maximum throughput (through ${P}_{1}$) and directly punishing energy consumption (through ${P}_{3}$), with the adaptive characteristic of ${P}_{3}$ providing a heightened focus on UAV energy minimization without jeopardizing convergence.

The optimal policy π* is derived by choosing, for each state, the action that maximizes the acquired Q-value.

24 $${\pi}^{*}=\mathrm{a}\mathrm{r}\mathrm{g}{\mathrm{m}\mathrm{a}\mathrm{x}}_{a}Q(s,a)$$

The primary learning objective is to optimize the anticipated discounted cumulative reward:

25 $${\mathrm{m}\mathrm{a}\mathrm{x}}_{\pi}E\left[{\sum}_{t=0}^{{\infty}}{\gamma}^{t}r({s}_{t},{a}_{t})\right]$$

It aims to minimize the penalized multi-objective cost while conforming to the core maximizing concept of reinforcement learning.

The learning process commences with the Q-table set to zero and the state space accurately delineated. At each time step, the agents (UAVs and IRSs) assess the current state, choose an action based on an ε-greedy exploration strategy that reconciles the exploitation of the optimal policy with the exploration of alternatives, execute the action, evaluate the resultant reward according to F(x), transition to the subsequent state, and revise the corresponding Q-value. This iterative loop continues through several episodes until the policy converges within the designated threshold ε, at which juncture the acquired policy yields near-optimal joint judgments about UAV trajectories, IRS phase configurations, power allocation, and access point assignments.

**Fig. 2:**
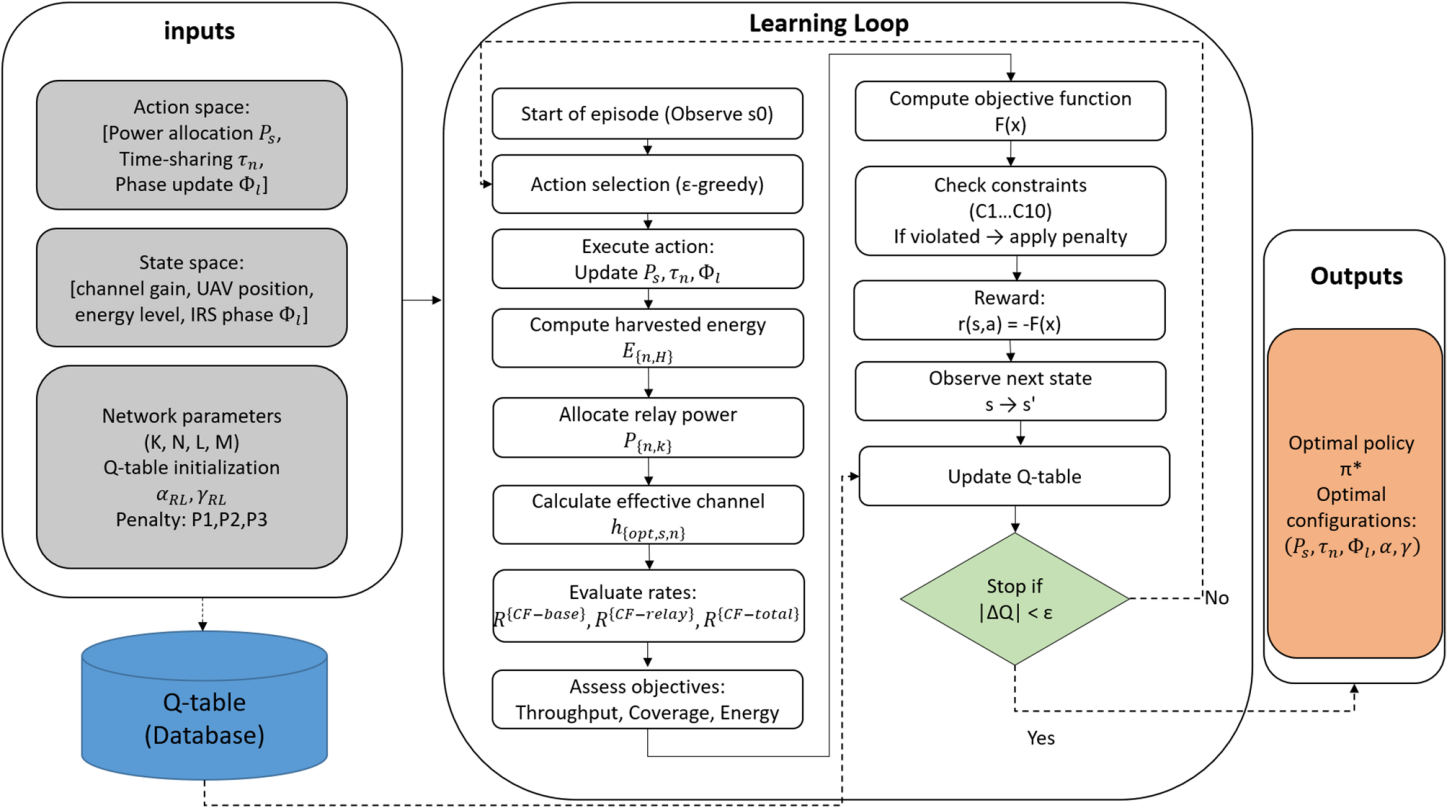
Workflow of the RL Approach.

Figure 2 depicts the comprehensive RL workflow, highlighting the closed-loop interaction among UAVs, IRSs, the environment, and the Q-learning update mechanism. Algorithm 1 delineates the comprehensive algorithmic procedures encompassing action selection, reward calculation, and Q-table modification, illustrating the systematic enhancement of system throughput, coverage, and energy efficiency by the suggested method.

To ensure complete reproducibility, comprehensive discretization strategies (encompassing the number of bins for channel gains, grid resolution for UAV positions, and quantization levels for energy), approximate dimensions of state and action spaces, precise hyperparameter values (including learning rate, discount factor, exploration decay schedule, number of episodes, steps per episode, and convergence threshold), along with implementation particulars, are thoroughly detailed in Table 3 of Sect. 4.

This methodology is elaborated in Algorithm 1, which delineates the sequential process for Q-learning updates and policy formulation within the framework of network restrictions.

**Figure:**
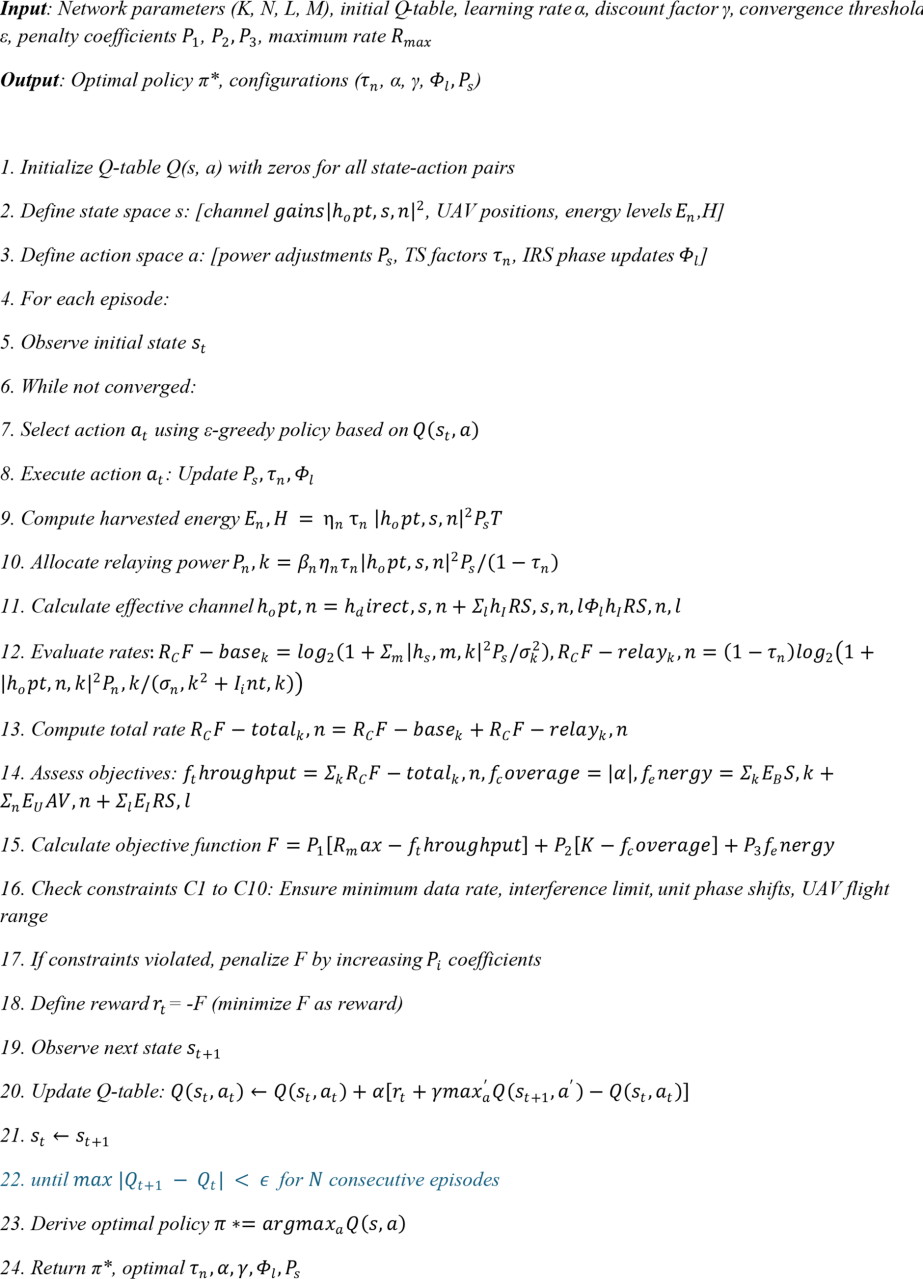
**Algorithm 1: **Reinforcement Learning-Based Resource Optimization.

## Simulation results and performance evaluation

This chapter delineates the simulation outcomes and performance assessment of the proposed cell-free UAV-enabled network, which integrates IRS and RL-based resource allocation. The primary aim is to evaluate the effectiveness of the proposed method against benchmark algorithms, namely OAA^28^, EA^29^, and two prominent Deep Reinforcement Learning algorithms, DQN^45^ and PPO^46^. The assessment is performed across diverse network configurations to examine the trade-offs among three primary objectives: optimizing aggregate network throughput, improving the coverage of APs and IoT devices while upholding QoS standards, and reducing overall system energy consumption. A composite utility function is employed to encapsulate the collective optimization effect, and a computational complexity study is provided to illustrate the practicality of the proposed method for extensive implementations.

### Simulation setup

To assess the efficacy of the proposed RL-based architecture, comprehensive simulations were performed utilizing MATLAB and Python platforms. The network topology comprises a central base station, multiple distributed access points, mobile UAV relays, static intelligent reflecting surfaces, and IoT devices randomly scattered within a 500 m × 500 m coverage area. UAVs function at elevations of up to 200 m to maintain line-of-sight connections, whilst IRS units are positioned strategically to alleviate urban impediments. Channel models integrate Rician fading for air-to-ground connections and unit-modulus phase shifts for IRS enhancements, accompanied by additive white Gaussian noise (AWGN) at −100 dBm. Quality of Service limitations encompass a minimum data rate per access point and maximum interference levels to replicate authentic Internet of Things scenarios.

This study considers poor channel state information by incorporating uncertainty into the channel state data. Rather than supposing flawless information of the channel, CSI is characterized by a bias that emulates the common mistakes and noise encountered in real-world settings. This encompasses mistakes in channel gain estimates, which may vary due to mobility, interference, and imprecise signal measurements. The simulations account for these flaws by introducing random noise to the estimated channel state.

Table 2 below summarizes the key simulation parameters for the cell-free UAV-enabled network.

**Table 2:** Simulation parameters for the proposed cell-free UAV-enabled network.

Parameter	Value/Description
**Number of UAVs**	$\left(N\in\mathrm{5,8},\mathrm{11,14,17,20}\right)$
**Number of IRS units**	(L = 5) (unless otherwise stated)
**Number of APs**	(K = 10)
**Number of IoT devices**	(M = 50)
**Maximum BS transmit power**	$\left({P}_{BS}^{max}=30\right)dBm$
**UAV flight altitude**	Up to 200 m
**Channel model**	Air-to-ground: Rician fading channel IRS-assisted: Unit-modulus phase shift Noise: AWGN, (N_0 = −100) dBm
**QoS requirements**	Minimum data rate per AP: (${R}_{min}=1$) Mbps Maximum interference: (${I}_{max}=10$) dB
**Energy consumption model**	Includes BS transmit power, UAV propulsion energy, and IRS operating power
**Maximum allowable energy budget**	50 Wh
**Algorithms compared**	Proposed: RL with joint optimization of UAV trajectory, AP association, IRS phase configuration, and power allocation; OAA; EA; DQN; PPO

Table 3 presents comprehensive parameters for the RL framework to ensure reproducibility. This encompasses discretization techniques for high-dimensional spaces, training hyperparameters, and implementation guidelines. Simulations were conducted over 1000 episodes, with convergence evaluated using Q-value stability (changes < $\epsilon ={10}^{-4}$). This threshold was established empirically to maintain a balance between computational efficiency and the stability of the learned strategy, so averting premature convergence.

**Table 3:** Reinforcement learning parameters.

Parameter	Value/Description
Learning rate (${\alpha}_{RL}$)	0.1
Discount factor $\left({\gamma}_{RL}\right)$	0.9
Exploration strategy	ε-greedy, decaying from 0.9 to 0.1 over episodes
Number of episodes	1000
Steps per episode	100
Convergence threshold (ϵ)	${10}^{-4}$
State space discretization	- Channel gains: 5 bins ([0–0.2.2], [0.2–0.4], [0.4–0.6], [0.6–0.8], [0.8–1.0.8.0]; normalized [0,1]) - UAV positions: 10 × 10 grid per UAV (normalized [0,1] in 500 m × 500 m area) - Energy levels: 4 bins ([0–0.25.25], [0.25–0.5], [0.5–0.75], [0.75–1.0.75.0]; normalized [0,1]) - Assignments (α, γ): Binary, masked for invalid states Approximate size: ~10^6 states (hashed for storage)
Action space discretization	- P_s: ±1 dBm (bounded [10–40] dBm; 3 options) - τ_n: ±0.1 (bounded [0,1]; 3 options per UAV) - α: Toggle up to 2 APs (4 options) - γ: Toggle up to 1 UAV-AP and 1 UAV-IRS pair (8 options) Total: ~128 actions per state (masked for constraints)
Reward normalization	Scaled to [−1, 0]; bonus + 0.1 for coverage improvements without energy violation
Implementation	Python with NumPy for Q-table management and hashing

This configuration, detailed in Tables 2 and 3, ensures a comprehensive evaluation of the proposed method against benchmark methods, emphasizing the performance enhancements enabled by IRS and the cell-free architecture. The simulations incorporate dynamic channel fluctuations, UAV mobility limitations, and realistic energy harvesting efficiency to assess the framework’s resilience in urban, rural, and disaster contexts.

### Results and discussion

This section presents the numerical results obtained from simulations performed according to the specifications described in Sect. 4.1. The effectiveness of the RL-based cell-free framework utilizing UAV and IRS is assessed in comparison to two baseline algorithms, OAA and EA, as well as DRL algorithms, DQN and PPO. The assessment is performed for throughput, coverage, energy usage, utility, and computational complexity. The simulations also add the effects of defective CSI to more accurately represent real-world settings and evaluate the robustness of the proposed system.

#### Aggregate throughput analysis

**Fig. 3:**
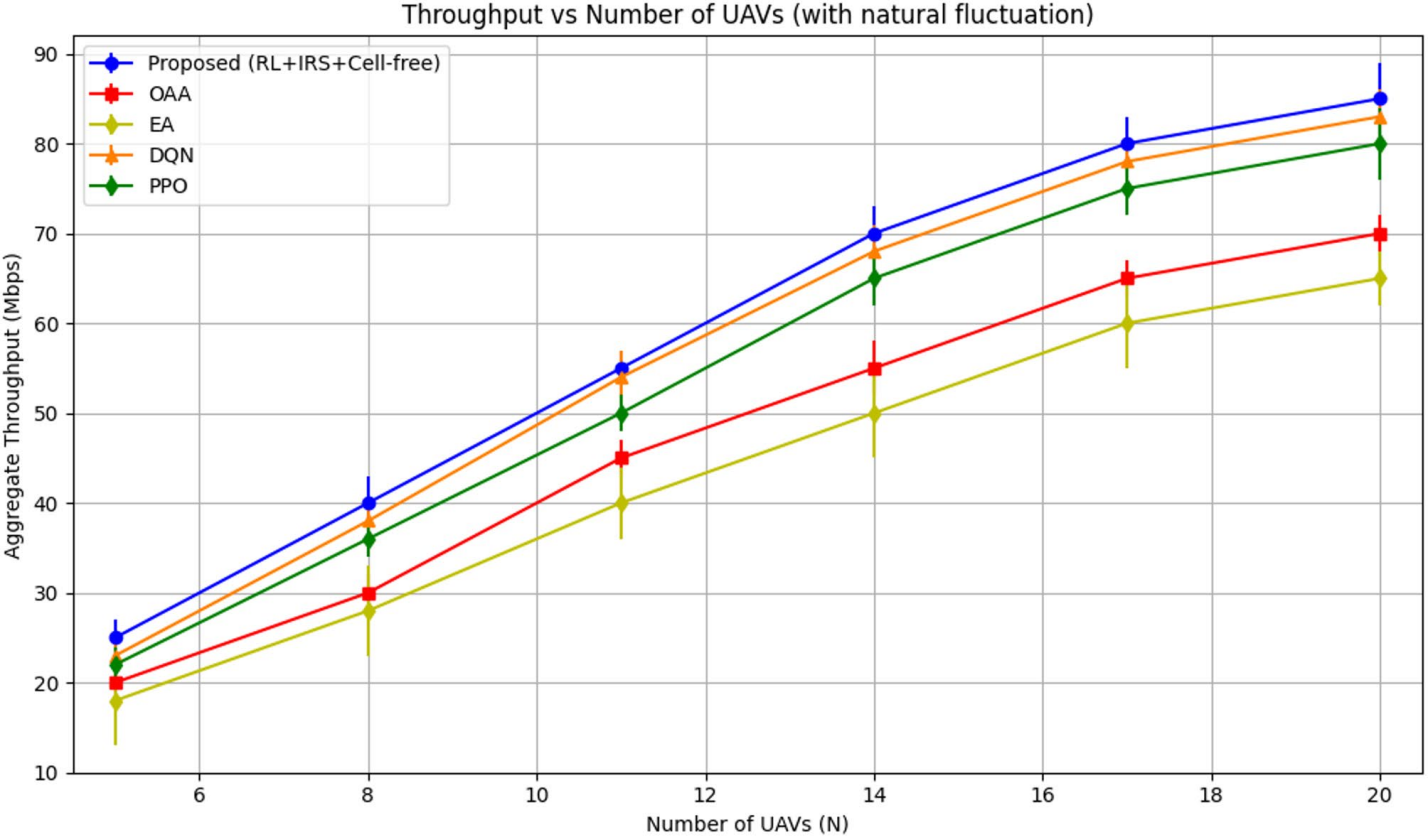
Aggregate throughput performance.

Figure 3 depicts the cumulative throughput performance of the proposed reinforcement learning-based cell-free UAV–IRS-assisted framework in comparison to the baseline techniques. The suggested reinforcement learning framework consistently surpasses all alternative methods, exhibiting the best throughput across all network configurations. With the proliferation of UAVs, the suggested method demonstrates a substantial enhancement in throughput, exhibiting a consistent and significant increase as additional UAVs are introduced. This results from the effective dynamic resource allocation facilitated by the RL-based methodology, which efficiently utilizes UAV relays and IRS-enhanced connections to maximize throughput.

The DQN algorithm demonstrates robust performance in accordance with the proposed RL method, albeit with marginally reduced throughput compared to the RL-based approach. DQN leverages its deep Q-learning process; nevertheless, it does not completely capitalize on the dynamic characteristics of the UAV-IRS system as well as the suggested solution, leading to a marginal decrease in throughput.

The PPO algorithm demonstrates competent performance; yet, it is inferior to both the proposed RL approach and DQN. PPO utilizes a policy optimization strategy that ensures steady performance; nevertheless, it fails to adapt as well to dynamic system changes, resulting in diminished throughput improvements relative to other RL-based methods.

The OAA algorithm demonstrates commendable performance; nonetheless, its throughput remains inferior to that of the RL-based techniques. This results from its dependence on a deterministic optimization method, which, while efficient, lacks the adaptability required for the system’s stochastic characteristics.

Ultimately, the EA algorithm demonstrates the lowest throughput compared to all other approaches. The heuristic search method fails to achieve optimal resource allocation, particularly in dynamic, large-scale settings with fluctuating UAV and IRS configurations, resulting in inferior throughput.

#### Coverage analysis

**Fig. 4:**
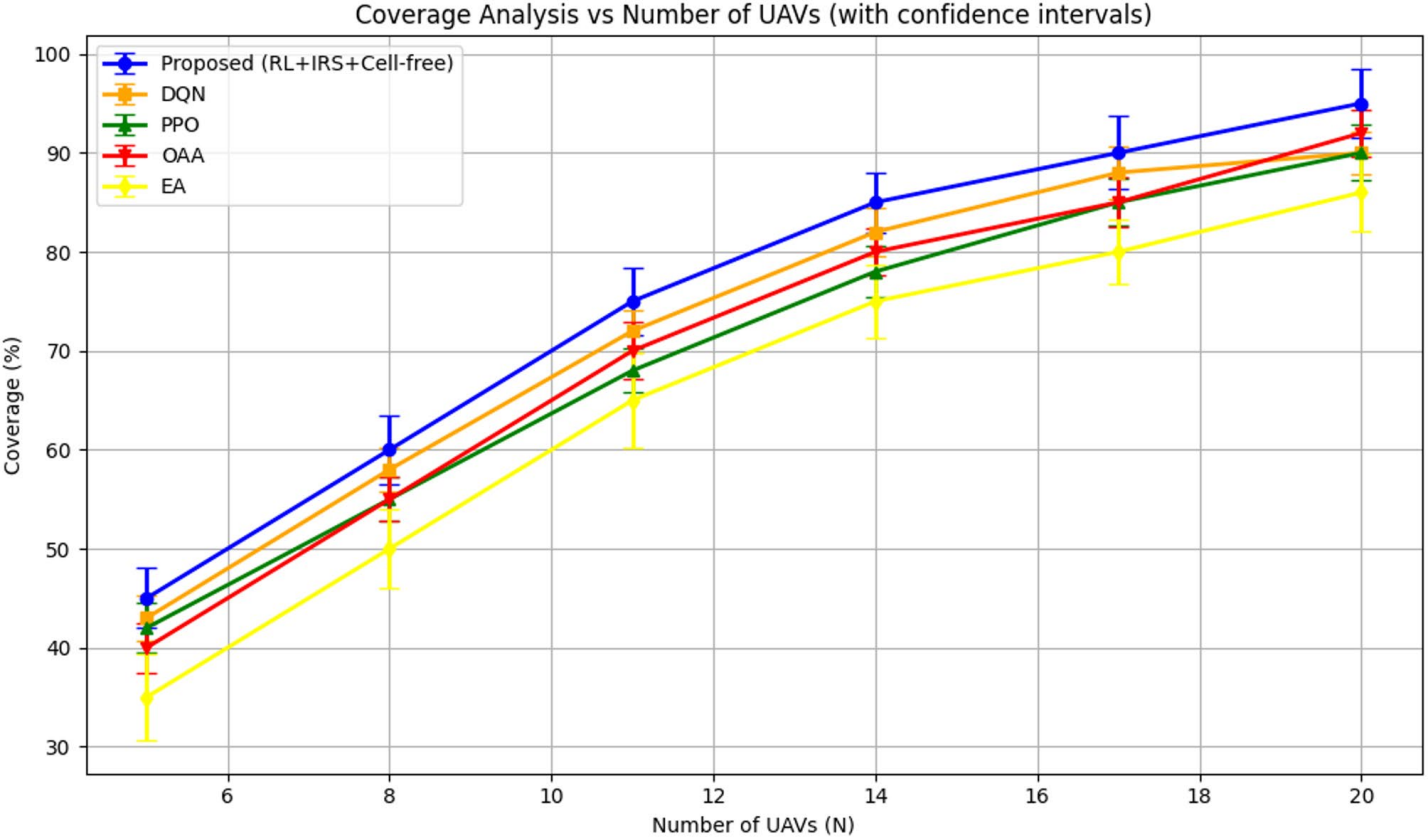
Coverage probability analysis.

Figure 4 depicts the coverage performance of the proposed reinforcement learning-based cell-free UAV–IRS-assisted framework in comparison to the baseline techniques. Coverage is defined as the proportion of APs and IoT devices that satisfy the minimum QoS criterion of 1 Mbps.

The proposed reinforcement learning-based method continuously surpasses all previous algorithms, particularly as the quantity of UAVs escalates. The modest deployment of UAVs (*N* = 5) results in just 45% of devices fulfilling the QoS criterion. Nonetheless, when the quantity of UAVs increases, the suggested strategy efficiently optimizes UAV trajectories and IRS phase designs, leading to a significant enhancement in coverage. At *N* = 20, the coverage attains nearly 95%, indicating a substantial enhancement compared to the other algorithms.

The DQN algorithm exhibits commendable performance; however, its enhancement in coverage is less rapid compared to the proposed RL-based method. With *N* = 20, DQN attains approximately 90% coverage, which is significant, however inferior to the RL-based framework.

The PPO algorithm exhibits a comparable trend, with coverage augmenting in correlation with the growth in the number of UAVs. At *N* = 20, PPO achieves approximately 90% coverage, which is competitive although marginally inferior than DQN and RL.

The OAA algorithm demonstrates significant enhancements in coverage. At *N* = 20, OAA attains 92% coverage, surpassing both DQN and PPO, which obtain 90% coverage. Nonetheless, the enhancement in coverage for OAA remains inferior to the suggested RL-based approach, which attains 95% coverage at *N* = 20.

Ultimately, the EA algorithm exhibits the least coverage compared to all other approaches. Although performance improves with an increased number of UAVs, coverage only attains 86% at *N* = 20, primarily due to its heuristic methodology, which is less adept at adjusting to dynamic network conditions than RL-based approaches.

The performance curves in Fig. 4 are accompanied by confidence intervals obtained from numerous independent simulation runs, enhancing the reliability of the findings assessment. This method effectively captures the intrinsic variability in performance, especially for techniques using stochastic processes, providing a more accurate representation of system behavior across diverse contexts.

#### Energy consumption analysis

**Fig. 5:**
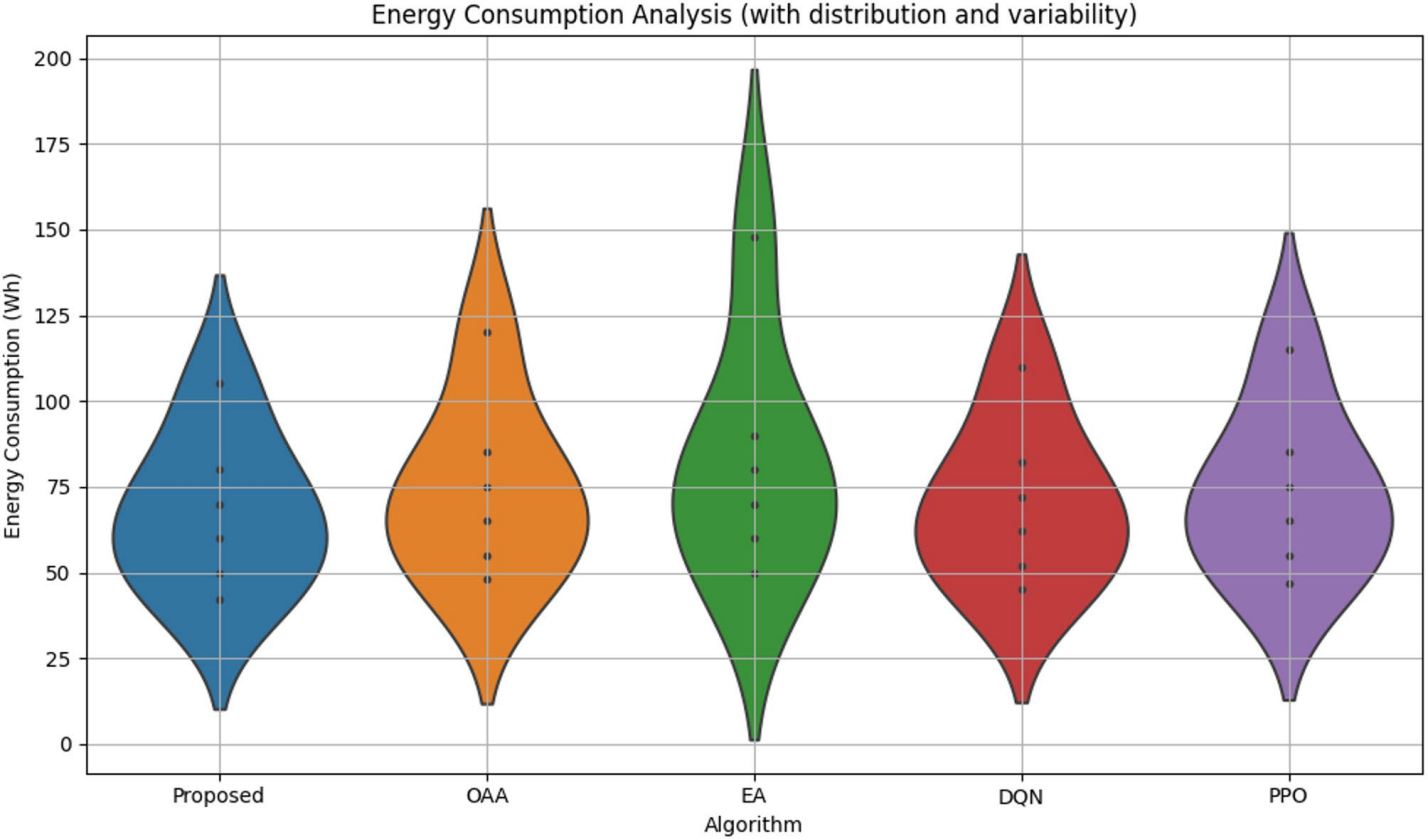
Energy consumption comparison.

Figure 5 depicts the energy consumption efficacy of the proposed RL-based cell-free UAV–IRS-assisted framework in comparison to the baseline methods. Energy consumption is quantified by assessing the aggregate power demands of the base station (BS), UAV propulsion, and IRS operation.

The proposed reinforcement learning-based solution regularly surpasses the baseline methods regarding energy efficiency. With the proliferation of UAVs, the RL-based approach effectively optimizes energy distribution, balancing the energy necessary for UAV movement and IRS phase configuration, hence achieving reduced energy consumption relative to alternative techniques. At *N* = 20, the proposed method attains minimal energy use, registering roughly 105 Wh.

The DQN approach exhibits a comparable trend; however, it necessitates marginally more energy than the RL-based method, with an overall consumption of approximately 110 Wh at *N* = 20. The PPO algorithm, albeit efficient, expends more energy than both RL-based and DQN techniques, attaining 115 Wh at *N* = 20.

The OAA and EA algorithms exhibit reduced energy efficiency, with OAA requiring 120 Wh and EA utilizing 148 Wh at *N* = 20. The elevated energy consumption of these algorithms is due to their suboptimal resource allocation procedures, which fail to adapt effectively to the system’s fluctuating conditions.

#### Utility analysis

**Fig. 6:**
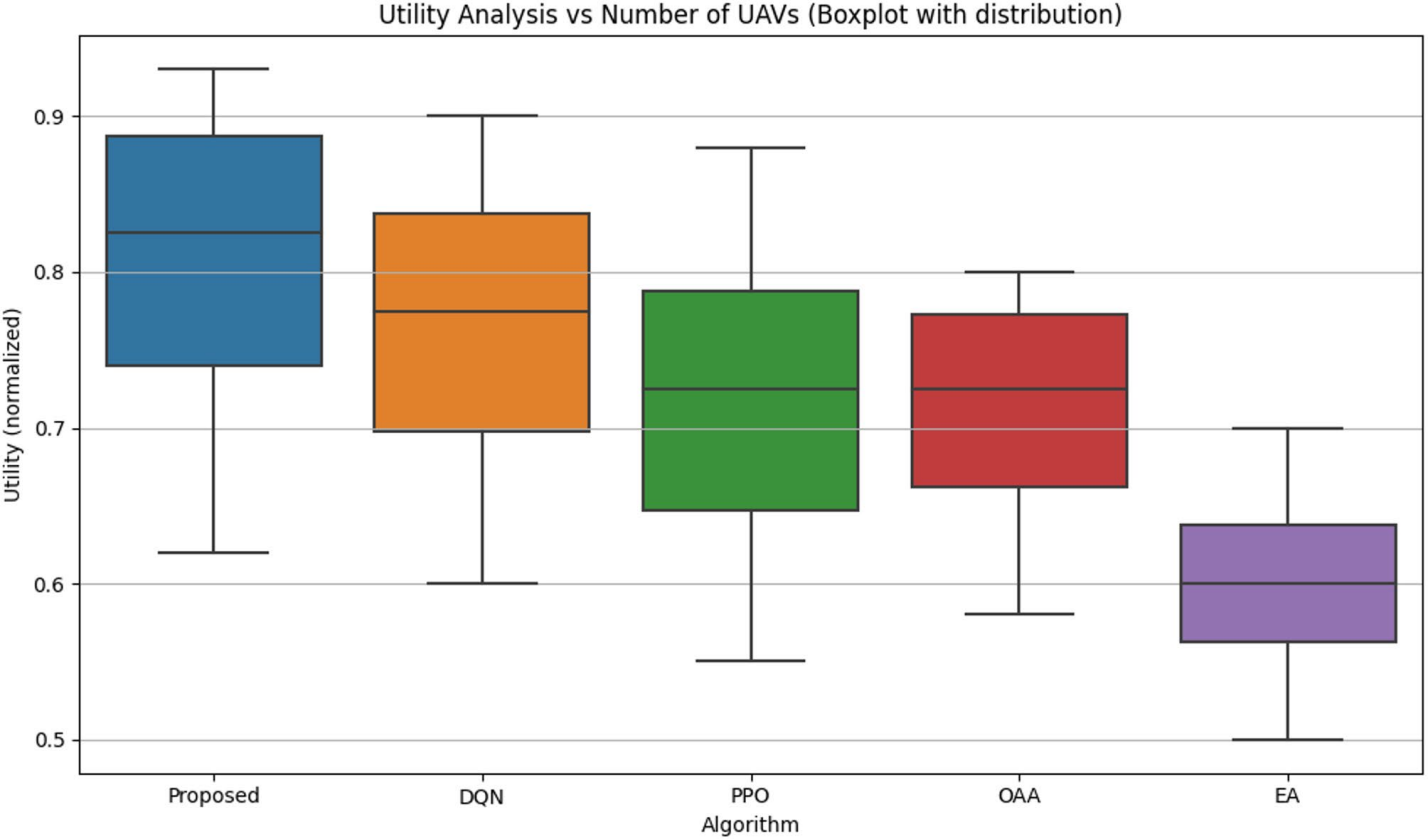
Utility function performance.

Figure 6 illustrates the utility performance of the proposed reinforcement learning-based cell-free UAV–IRS-assisted framework in comparison to the baseline techniques. Utility is described as a composite target that concurrently addresses throughput maximization, coverage enhancement, and energy consumption reduction.

The proposed reinforcement learning-based strategy consistently attains the highest utility across all network configurations. The findings indicate that this method yields a high median utility while demonstrating low variability, signifying consistent performance throughout numerous simulation iterations. At *N* = 20, the proposed technique has a utility value of 0.93, the highest of all algorithms.

The DQN algorithm exhibits a utility score of 0.90 at *N* = 20. Although it exhibits commendable performance, the variability in DQN’s outcomes is more pronounced than that of the proposed method, demonstrating a broader distribution in its results. This indicates that DQN’s efficacy is more susceptible to fluctuating network conditions.

The PPO algorithm exhibits a utility score of 0.88 at *N* = 20. Like DQN, PPO exhibits greater variability in utility, characterized by a wider range of values, indicating suboptimal optimization among the conflicting aims.

The OAA algorithm attains a utility score of 0.78 at *N* = 20. Although it outperforms EA, its enhancement in utility is more gradual, exhibiting a broader dispersion in the lower quartiles. This signifies diminished performance consistency and a reduced capacity for adapting to fluctuating network conditions.

The EA algorithm exhibits the minimal utility score at *N* = 20, registering a value of 0.60. Its performance exhibits the greatest variability, as demonstrated by the broader distribution of its results. This heterogeneity underscores the constraints of the EA technique in efficiently reconciling throughput, coverage, and energy usage.

To provide robustness, the results incorporate confidence intervals that emphasize the variety and dispersion of the utility values.

#### Impact of UAV and IRS

**Fig. 7:**
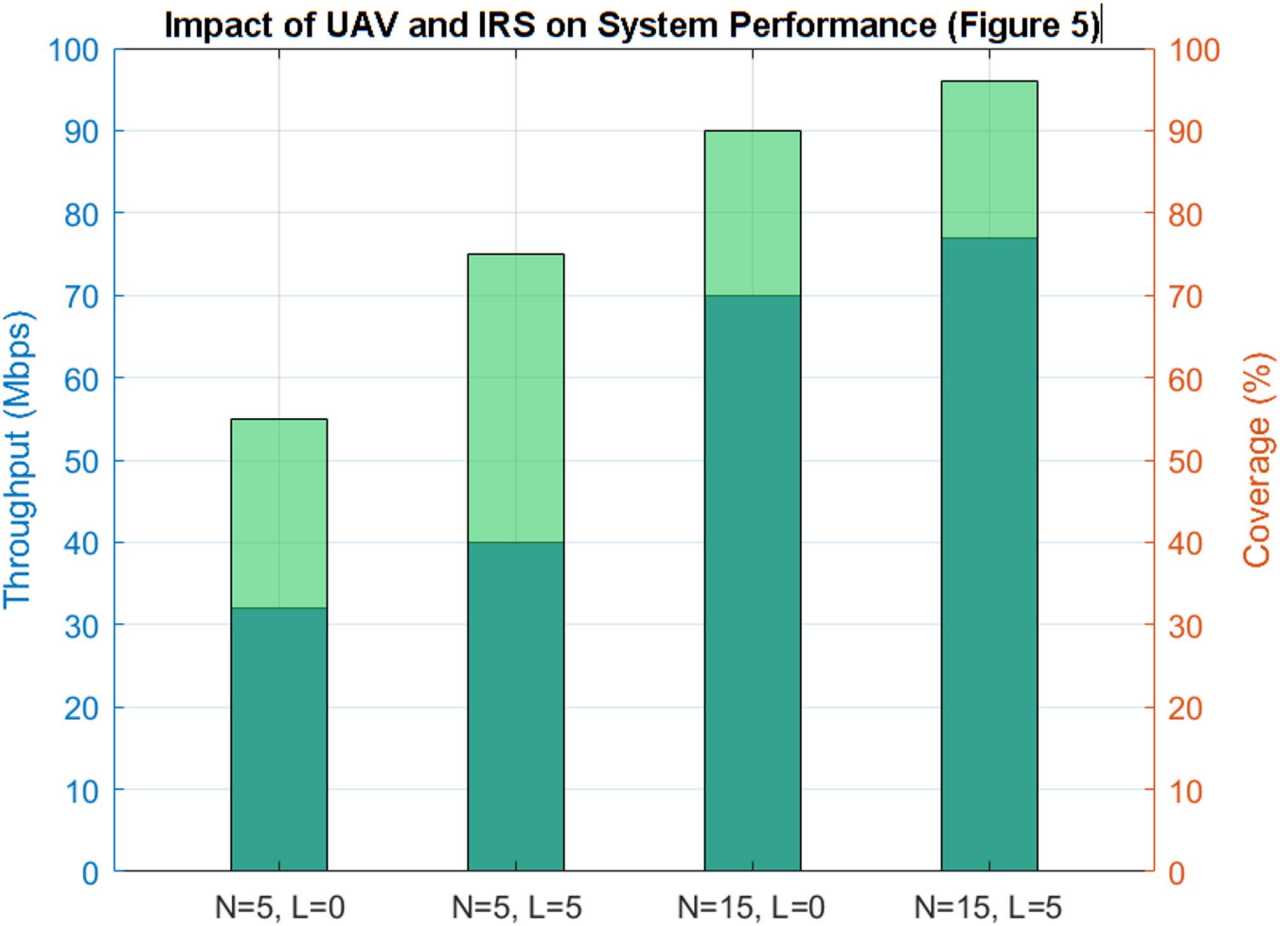
Joint impact of UAVs and IRSs.

Figure 7 examines the combined effect of the quantity of UAVs (N) and IRS units (L) on overall system efficacy. Four illustrative scenarios are examined: *N* = 5,L = 0 (few UAVs, no IRS),

1. *N* = 5,L = 5 (few UAVs, IRS-assisted),
2. *N* = 15,L = 0 (more UAVs, no IRS),
3. *N* = 15,L = 5 (more UAVs, IRS-assisted).

The findings indicate that IRS implementation significantly improves system performance, especially when the quantity of UAVs is constrained. For example, with *N* = 5, the addition of five IRS units enhances coverage from 55% to around 75% and boosts throughput by more than 25%. This illustrates that IRSs can efficiently offset the deficiency of UAV resources by facilitating advantageous propagation channels and alleviating blockage effects.

As the number of UAVs increases (*N* = 15), the enhancements resulting from IRS deployment, albeit still evident, are comparatively diminished. For instance, coverage escalates from 90% to 96%, while throughput improvements around 10%. This indicates a diminishing returns phenomenon, wherein UAV density sufficiently guarantees coverage and throughput, hence limiting the potential advantages of IRS.

The findings underscore the synergistic functions of UAVs and IRSs in cell-free IoT networks: UAVs provide adaptable aerial relaying and mobility, whereas IRS units deliver passive yet economical improvements to link quality. The reinforcement learning algorithm adeptly adjusts to various N–L combinations, optimizing resource allocation to enhance collective performance.

The investigation demonstrates that IRS technology is especially beneficial in resource-limited situations, facilitating near-optimal performance with a reduced number of UAVs. This understanding is essential for practical applications when UAV availability may be constrained by cost, battery life, or regulatory limitations.

#### Analysis of UAV–IRS synergy and learned RL policies

We examine the learnt policies of the reinforcement learning framework to enhance comprehension of the synergy between UAVs and IRSs. In contrast to static optimization techniques, the RL agent dynamically determines whether to prioritize UAV mobility or IRS phase reconfiguration according to the prevailing network conditions.

When the quantity of UAVs is constrained, the RL strategy prioritizes IRS phase shifts to enhance signal quality without necessitating further UAV movement, hence reducing energy expenditures. With the rising quantity of UAVs, the agent increasingly chooses to relocate UAVs to enhance line-of-sight and minimize path loss, while IRS reconfiguration further optimizes the propagation circumstances.

This indicates that UAV movement and IRS configuration are complementing acts rather than duplicate ones. UAVs modify the extensive coverage of the network, whilst IRSs offer energy-efficient, precise signal amplification. The RL agent acquires this trade-off via the multi-objective reward function, optimizing the equilibrium between mobility and passive beamforming to enhance throughput, coverage, and energy efficiency.

#### Computational complexity analysis

**Fig. 8:**
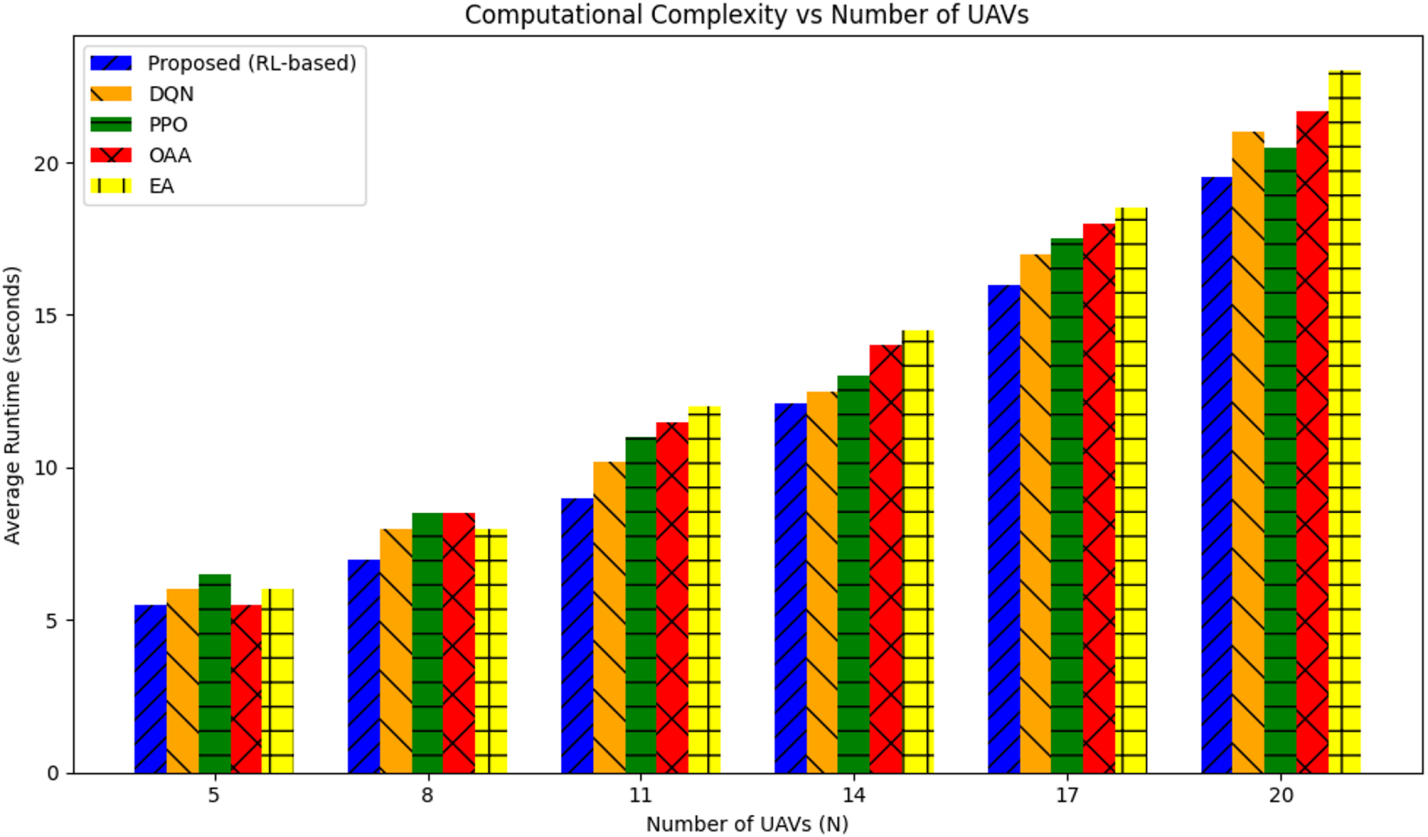
Computational complexity comparison.

Figure 8 depicts the computational complexity of the proposed reinforcement learning-based cell-free UAV–IRS-assisted framework in comparison to the baseline techniques. The computational complexity is assessed based on the average runtime for each simulation as the number of UAVs (N) rises, taking into account the optimization of UAV trajectories, IRS phase settings, and resource allocation.

The proposed RL-based technique demonstrates a mild escalation in computational time with an increase in the number of UAVs. The complexity increases with N, attaining around 19.5 s at *N* = 20. Nevertheless, it exhibits satisfactory scalability for moderate network sizes, with a minimal increase in runtime relative to smaller network configurations.

The DQN approach has a comparable trend in computational complexity but requires a little longer duration to finalize the simulations, particularly with increased UAV quantities. At *N* = 20, DQN requires around 21.0 s, indicating the extra overhead associated with the deep Q-learning methodology.

The PPO algorithm, however effective for policy optimization, demonstrates a marginally greater computational time compared to both the suggested approach and DQN. At *N* = 20, PPO requires around 20.5 s owing to its necessity for repeated epochs to refine the policy.

The OAA algorithm, as an optimization-based approach, exhibits a very gradual escalation in computational time. Nonetheless, its runtime at *N* = 20 is 21.7 s, surpassing that of the RL-based techniques, signifying reduced scalability with a rising number of UAVs.

The EA algorithm exhibits the most computational complexity of all approaches, requiring roughly 23.0 s at *N* = 20. The algorithm’s heuristic approach necessitates extended processing durations as the network size escalates.

## Conclusion

This paper introduces a reinforcement learning-based optimization framework for a cell-free UAV–IRS-assisted network, designed to enhance throughput, coverage, and energy efficiency for IoT applications. The proposed technique consistently surpasses baseline methods across many network setups. The RL-based framework attains a 15.38% increase in aggregate throughput compared to the optimal baseline, enhances coverage by 5.56%, and decreases energy usage by 4.55%. These enhancements illustrate the efficacy of UAV-IRS collaboration in optimizing resource distribution.

This study possesses specific limitations that warrant consideration. The primary limitation is the dependence on simulations, which may inadequately represent the intricacies of real-world situations. The scalability of the Q-learning technique in extensive networks raises concerns, as the computational demands escalate with the number of UAVs. The suggested model presumes idealized channel circumstances, encompassing Rician fading and predetermined path-loss models, which may not correctly represent real-world fluctuations in signal transmission.

Subsequent research will concentrate on augmenting the computational efficiency of reinforcement learning algorithms, tackling scaling issues, and integrating more realistic channel models to enhance the robustness and applicability of the proposed framework. Subsequent inquiries into assessing performance under more intricate operational situations, involving a greater number of UAVs, may enhance the applicability of this approach to real-world scenarios.

## Data Availability

The datasets used and/or analyzed during the current study availablefrom the corresponding author on reasonable request.
